# Advancements in Fabrication and Application of Chitosan Composites in Implants and Dentistry: A Review

**DOI:** 10.3390/biom12020155

**Published:** 2022-01-18

**Authors:** Fariborz Sharifianjazi, Samad Khaksar, Amirhossein Esmaeilkhanian, Leila Bazli, Sara Eskandarinezhad, Peyman Salahshour, Farnaz Sadeghi, Sadegh Rostamnia, Seyed Mohammad Vahdat

**Affiliations:** 1 School of Science and Technology, The University of Georgia, Tbilisi, Georgia; f.sharifianjazi@ug.edu.ge (F.S.); peyman_chemistry@yahoo.com (P.S.); 2 Department of Materials and Metallurgical Engineering, Amirkabir University of Technology, Tehran 1599637111, Iran; Esmaeilkhanian-a@aut.ac.ir; 3 School of Metallurgy and Materials Engineering, Iran University of Science and Technology (IUST), Tehran 16845-161, Iran; leilabazli64@gmail.com; 4 Department of Mining and Metallurgy, Yazd University, Yazd 89158-18411, Iran; s.eskandari.nezhad@gmail.com; 5 Department of Biomedical Engineering, Islamic Azad University of Yazd, Yazd 89158-13135, Iran; farnazsadeghi487@yahoo.com; 6 Organic and Nano Group (ONG), Department of Chemistry, Iran University of Science and Technology (IUST), Tehran 16846-13114, Iran; rostamnia@iust.ac.ir; 7 Department of Chemistry, Ayatollah Amoli Branch, Islamic Azad University, Amol 57169-63896, Iran; vahdat_mohammad@yahoo.com

**Keywords:** chitosan composite, implantable biomaterials, dentistry, dental engineering

## Abstract

Chitosan is a biopolymer that is found in nature and is produced from chitin deacetylation. Chitosan has been studied thoroughly for multiple applications with an interdisciplinary approach. Antifungal antibacterial activities, mucoadhesion, non-toxicity, biodegradability, and biocompatibility are some of the unique characteristics of chitosan-based biomaterials. Moreover, chitosan is the only widely-used natural polysaccharide, and it is possible to chemically modify it for different applications and functions. In various fields, chitosan composite and compound manufacturing has acquired much interest in developing several promising products. Chitosan and its derivatives have gained attention universally in biomedical and pharmaceutical industries as a result of their desired characteristics. In the present mini-review, novel methods for preparing chitosan-containing materials for dental and implant engineering applications along with challenges and future perspectives are discussed.

## 1. Introduction

A composite substance is a substance that is produced from two or more constituent substances that have distinctly different chemical or physical properties and combine to form a substance with properties different from those of individual elements [1,2,3]. Significant features have been observed in nano-composites that were generated using nanoparticle reinforcement under 100 nm. The environmental concerns due to the slow decomposition of nano-composite and composite materials are the major problem related to the application of these materials. A solution to this problem is producing composite materials from sources that are naturally found in nature [4,5,6,7].

There are synthetic and natural materials that have been applied for tissue engineering, such as polycaprolactone (PCL), poly (lactic-co-glycolic) acid (PGLA), polylactic acid (PLA), polyethylene glycol (PEG), polyvinyl alcohol (PVA), polyacrylamide, hyaluronic acid (HA), hydroxyapatite (HAp), silk fibroin, alginate (Alg), gelatin (GL), collagen, and chitosan (CS) [4,8,9,10,11,12,13,14,15,16,17,18,19].

Chitosan, with chemical formula of (C_6_H_11_O_4_N)_n_ is a deacetylate chitin derivative. The free amino groups of chitosan enable it to chemically bond to DNA, RNA, cholesterol, fat, proteins, and metal ions [20]. Chitosan also exhibits insolubility in most solvents but it is soluble in 1% hydrochloric acid and dilute nitric acid [21]. The main parameters directly affect its properties are degree of deacetylation (DD), molecular weight (Mw), ash content, and moisture [22,23].

Chitosan-HAp broadly has been studied because of its distinctive characteristics as a potential bioactive material. Partially deacetylation of the chitin from the crustacean exoskeleton yields chitosan [24,25,26]. Some of the chitosan’s properties are biological activity, antimicrobial activity, hydrophilicity, biocompatibility, and biodegradability. Also, chitosan has various techniques of processing such as microparticles, microspheres, tablets, paste, gel, film, sponge, blend, and solution. Hence, chitosan possesses various biomedical applications such as anti-infection, bone regeneration, dosing, tissue regeneration, drug delivery, and wound healing [20,27,28,29,30,31,32]. Chitosan is both cost-effective and an economical natural biopolymer since it is derived from renewable and natural resources.

For instance, chitosan promotes bone formation in bone tissue by boosting osteoblasts formation in addition to its ability to regenerate the connective tissue [33]. Moreover, it acts as a stimulant for the immune system as well as biomedical implementation for the central nervous system [34]. It also influences reducing blood pressure, regulating liver functions and the gastrointestinal system, and intestinal motility. Chitosan is similar to N-glycosaminoglycans, which are essential components of the connective tissues. Thus, it helps the 3D growth of the tissue matrix. Additionally, chitosan is favorable for fabricating coatings. Random calcium phosphate crystals formation with various morphologies and phases are promoted by chitosan scaffolds [35,36,37,38,39,40,41,42].

In addition, the cationic characteristic of chitosan offers a special chance for producing linear polyelectrolytes by combining them with surfaces with a negative charge like anionic polysaccharides and proteins. Composites of calcium phosphate and chitosan promote osteoblasts’ proliferation, and as a result, they are employed in bone tissue engineering. Many studies were performed to investigate the bioactivity of chitosan by the introduction of compounds that contain active functional groups [43]. For instance, the nanoparticles of (d, l-lactide-co-glycolide acid) PLGA-lovastatin-chitosan-tetracycline were used by YP et al. [44], showed enhanced alkaline phosphatase activity, superb osteogenic potential, antibacterial activity, high biocompatibility, and well-controlled release of drugs. In addition, forming bone was shown in the defects by micro-CT images, which were filled by nanoparticles [38,45].

Chitosan can be used in various application including the following:

(a) Wastewater treatment [46], water engineering [47], in which, it can act as a clotting agent and can also trap heavy metal ions as a chelating agent due to its polycationic nature.
(b) Paper and packaging industries [48], due to its biodegradability and high compatibility in the environment, the textile industry [49] due to its non-toxicity, biodegradablity and environmentally friendly nature.
(c) The food industry, as an additive in meat and dairy products due to its antimicrobial and antioxidant properties, as well as to prevent taste change and increase shelf life [50,51].
(d) Agriculture, due to the creation of a thin coating on fruits and vegetables, which acts as a protective film preventing spoilage of agricultural products [51].
(e) Medicine and biomedicine, depending on the chitosan purity it can be used in drug release and release systems, hemodialysis, artificial skin, linoleum, enzymatic immobilization, contact lenses, eye bandages, orthopedics, flossing, dentistry [52].
(f) Medical engineering like wound healing, tissue engineering, manufacture of drug carriers, chitosan nanocarriers for anticancer drugs and vaccine release, gene therapy and bioimaging of vital organs [53].

Many investigations have been carried out in dentistry that used chitosan to avoid the process of tooth decay since chitosan is capable of eliminating bacteria and/or exhibiting bacteriostatic characteristics. It was observed that chitosan with low molecular weight is able to prevent *Streptococcus mutans* from adsorbing to HAp. Moreover, chitosan is a bio-adhesive polymer, able to sustain on oral mucosa for longer periods [44,54]. Additionally, chitosan can be easily chemically modified with lower inflammatory response and great flexibility and elasticity properties, due to its β-(1,4) glycosidic bonds among D-glucosamine and N-acetyl-D-glucosamine. There have been numerous studies that reported fabricating scaffolds with biomedical applications by using a combination of chitosan and synthetic and natural materials. To enhance the integrity of the structure and mechanical strength of biomaterials that are based on chitosan, bioactive Nanoceramics like ZrO_2_, TiO_2_, SiO_2_, and HAp, and biopolymers like HA, PLA, PCL, GL, Alg, silk, and chitin were added to the chitosan-based biomaterial [37,55,56,57].

To our best knowledge, most of the review articles on chitosan have focused and reported on chitosan applications in biomedicine while none has discussed the chitosan composite applications progress in the fields of implant scaffolds and dentistry. As the rapid growth of attention has been attracted in this field, the present review aims to study chitosan compositions throughout the previous decade in dental engineering and implanted scaffolds. First, a summary of chitosan characteristics and structure, and methods of chitosan composite extractions is presented. Next, we demonstrate a comprehensive overview of the last methods for chitosan compositions synthesis. Finally, advances in chitosan composites in implants and dentistry fields are discussed.

## 2. Chitosan Composites Used in Implants and Dentistry

Chitosan possesses several desirable characteristics. These properties make chitosan a noteworthy biomaterial for various applications. Although, existing limitations like stability in aqueous solutions and mechanical features have put some restrictions on its uses in bone tissue engineering. In [58], pure scaffolds of chitosan have been successfully manufactured; however, many studies have been conducted to eliminate the restrictions related to pure implants of chitosan by combining chitosan with additives, carbon-based materials, or polymers [59,60,61].

Currently, composites are playing a significant role in dental and implant materials. Nowadays, chitosan composite materials and their applications in the fields of dental and implant engineering have gained a significant amount of interest owing to their biodegradability, biocompatibility, intrinsic antibacterial nature, and minimal reactions with a foreign body. Additionally, they can be molded into different formations and geometries like porous structures that all these properties have made desirable for osteoconduction and cell culture. Thus, chitosan composites are presented as candidate materials for bone regeneration and artificial bone in tissue engineering, and as well as applications in dentistry fields [58,62,63,64].

### 2.1. Composites of Chitosan/Carbon-Based Materials

**Figure 1:**
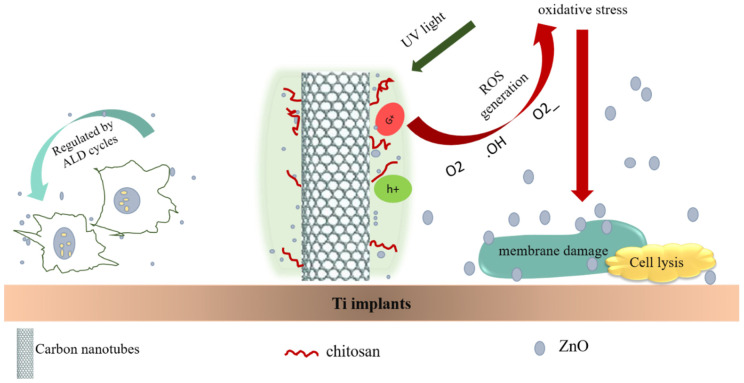
ZnO/chitosan/carbon nanotubes hybrid system biofunctionalization mechanism on Ti implants.

Carbon-based nanomaterials (CNMs), including graphene oxide (GO), graphene, and carbon nanotubes, are known as analog shapes due to their similar dimensions to the physical properties of extracellular matrix (ECM) components such as collagen fibers [65]. As a result of their remarkable mechanical features, these CNMs play an important role in strengthened inorganic/organic synthetic scaffolds, which would be favorable for applications in bone tissue engineering. Besides mechanical strength, CNMs offer electrical stimulation, which is employed in nerve tissue engineering. As CNMs show antibacterial activity due to differentiation into neural or osteo lineages, proliferation, induce adhesion of cell, and free π electron existence, they can be excellent materials to be applied in wound healing. Therefore, chitosan/CNM composite materials have gained significant attention. For instance, 1D nanostructures of ZnO on Ti modified with CNTs/CS were prepared by Zhu et al. (Figure 1). First, oxygenous groups were introduced onto the CNT surface to enhance the CNT biocompatibilities. Next, they were grafted with CS [66,67,68,69,70]. When the content of ZnO is insufficient, the antibacterial activity vs. *S. aureus* and *E. coli* can be improved by CNTs for 39% and 8%, respectively [71,72].

Montazeri and Karjibani devised the novel hydrogels of chitosan/double-walled carbon nanotubes (DWCNT) and chitosan/DWCNT-COOH. The enhanced hydrogels showed a distinct porous structure along with excellent uptake of water. The capacity of chitosan/DWCNT–COOH hydrogel’s water uptake was enhanced by introducing carboxyl groups. Also, the scattering ability of DWCNT-COOH in the chitosan matrix was improved by topological hindrance between carbon nanotubes. Additionally, compared to other hydrogels, superior scattering of DWCNTs enhanced the thermal stability of chitosan [73]. In a recent study conducted by Amiryaghoubi et al., a thermosensitive injectable hydrogel was prepared using natural polymer, chitosan, with different feed ratios via a composite of copolymer/GO based on poly (N-isopropyl acrylamide) (PNIPAAm) as physical and chemical crosslinking. These hydrogels were used for the osteoblast differentiation and proliferation of the human dental pulp stem cells (hDPSCs). The preparation of the composite of PNIPAAm copolymer/GO composite was performed through free-radical copolymerization of (N-isopropyl acrylamide) (NIPAAm), itaconic acid (IA), and maleic anhydride-modified poly (ethylene glycol) (PEG) in the presence of GO and applied for the hydrogel fabrication [8,73,74].

The alkaline phosphatase (ALP) activity and deposition of minerals can be improved by hydrogel, as a result of the amine and oxygen-including CS and GO functional groups. The tailored hydrogel could up-regulate the introduction of Runt-related transcription factor 2 and osteocalcin into the hDPSCs that cultivated in both normal and osteogenic media. It looks also for the promotion of osteogenic inducers absorption. Moreover, a composite coating of graphene oxide/chitosan/hydroxyapatite (GO/CS/HAp) was prepared via electrophoretic deposition on the Ti substrates. Ti was coated by a crack-free and homogeneous composite coating of GO/CS/HAp. Furthermore, the strength of bonding and wettability of coating on the GO/CS/HAp composite were improved in comparison with HA, GO/HAp, and CS/HAp coatings. Additionally, the coating of GO/CS/HAp highly increased the cell-material interactions in vitro. Moreover, this GO/CS/HAp coating on the Ti implant develops osseointegration in vivo. As a result, GO/CS/HAp -Ti can be applied in the dental implant field [75,76].

### 2.2. Composites of Chitosan/Polymer-Based Materials

Considerable research has been done on mixing natural polymers with synthetic polymers. For instance, chitosan is usually combined with hydrophobic synthetic polymers, decreasing the chitosan hydrophilic characteristics and enhancing its mechanical properties. A diverse range of synthetic polymers has been studied for bone applications, including PCL, poly (lactic acid) (PLA), polyglycolic acid (PGA), and poly (lactic-co-glycolic acid) PLGA. The composition of PLGA and chitosan has been studied for bone tissue engineering. This composition mainly includes PLGA microspheres/nanoparticles loaded with growth factors/drugs that merged in implants containing chitosan to apply as a drug delivery system including bioactive molecule or local delivery. Moreover, to prepare scaffolds with porous structure, PLGA microspheres and chitosan have also been employed [77,78,79].

The chitosan-PLGA scaffolds compared to other PLGA-based composites, show significant osteogenesis as well as cellular compatibility. A 3D printed scaffold composed of chitosan, gelatin, and HAp was developed by Chen et al. [78] to repair the critical-sized defects of calvarial bone in the rabbit model. The developed scaffolds exhibited a structure with inter-connective pores and by a simple adsorption way it was able to load BMP-2 and VEGF. As the cell culture results indicate the scaffolds are ideal candidates for osteogenic differentiation and proliferation of MC3T3-E1 cells. Especially, the additive influence of developing MC3T3-E1 cells osteogenic differentiation by increasing activity of ALP up-regulation of OPN and Col I introduction as indicated in growth factors of scaffolds. In the defect model of rabbit calvarial bone, the scaffolds with growth factors exhibited a favorable new bone manufacturing through enhancing regeneration of blood vessels and bone. As mentioned, these kinds of composite scaffolds are the most desirable scaffolds for bone tissue engineering [77].

**Figure 2:**
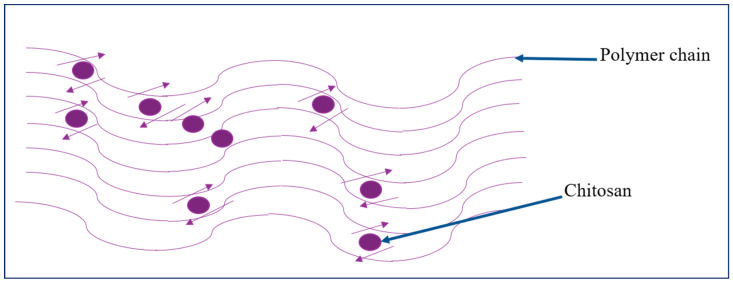
Schematic representation of the slippage of the polymeric chain on/around chitosan particles.

Singh et al. [80] reported the preparation of chitosan-reinforced PLA scaffolds through fused filament fabrication (FFF) 3D printing technology (shown in Figure 2). From the results from the mechanical characterization through statistical analysis the following can be concluded: by increasing chitosan weight fraction, the tensile strength reduces. This was because of the introduction of discontinuities in the polymeric chains by the existence of chitosan particles and also chain slippages on the chitosan. Raising infill density make higher tensile strength (TS). Due to the higher density of material composition, compressive strength is enhanced by the weight fraction of chitosan.

### 2.3. Composites of Chitosan/Metal or Metal Oxides

The most advantageous characteristic of chitosan is that chitosan is able to chelate with a wide range of metal ions, specifically transition ones. Furthermore, in order to strengthen the bond between restorative materials metal oxides like silica, zirconia, and alumina are mostly used. In addition, chitosan nanoparticles have demonstrated antibacterial activity against a broad range of bacteria. Moreover, chitosan addition to composites improves their antibacterial features without disturbing their shear bond strength (SBS). For example, in a study by Gawad et al., employing a new cobalt and chitosan NPs coating and a novel calcium hydrogen phosphate drug assisting the bone treatment procedure was investigated. The results from this study were in accordance with that the low-cost surgical 316L SS alloy nanocoated with CaHP drug that aids the bone formation, being suitable as a human body’s bone implant. Thus, it can be utilized for biomedical applications. A green fabrication approach for Au-chitosan hNPs synthesis with the combination of chitosan as a reducing and capping agent was introduced by Mohamady Hossein et al. For the first time, the influence of chitosan concentration on the physicochemical and antimicrobial properties of Au-chitosan hNPs by using very low chitosan concentrations was studied. Most of the results presented that the Au-chitosan hNP sizes are inversely proportional to the chitosan amount that is employed in the synthesis technique [81,82,83,84,85,86].

**Figure 3:**
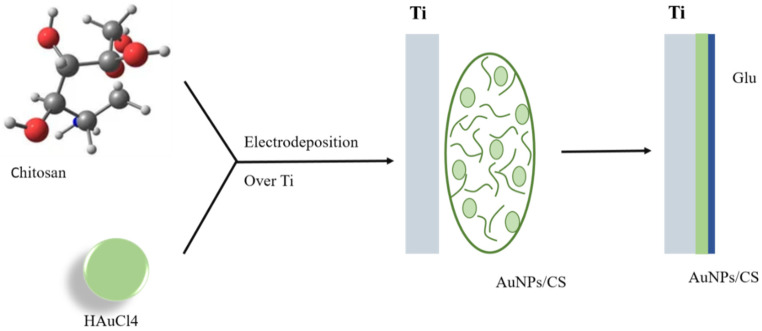
Schematic view of the bio nanocomposite coat from the electrodeposited bio nanocomposite film growth mechanism on the surface of Ti substrate.

However, enhancing the chitosan amount increased the positive potential. Additionally, the antimicrobial activity of the fabricated Au-CS hNPs in the analysis of Colony Forming Unit (CFU) and Minimum Inhibitory Concentration (MIC) was improved by increasing chitosan amount due to the fact that antibacterial activity was credited to the positive potential and small size that were acquired with the highest chitosan concentration. Moreover, the Au-chitosan hNPs containing the highest amount of chitosan exhibited the best thermal stability. Furthermore, the effect of AuNPs/CS bio nanocomposite film on corrosion resistance of Ti and fabricated extremely uniform bio nanocomposite film combined with CS and gold nanoparticles (AuNPs) using electrodeposition technique (Figure 3) was studied by Farghali et al. The highly efficient bio nanocomposite film rendered the antibacterial impact to avoid bacterial growth. Moreover, using solvent casting procedure, the composite containing three components of chitosan/polyvinyl pyrrolidone (CS/PVP) was synthesized in various weight ratios (0, 20, 40, 60, and 80 wt.%) of copper–hydroxyapatite (Cu-HAp). Blood compatibility of Cu-HAp/CS/PVP compositions was proved by in vitro hemocompatibility survey. With a hemolytic ratio of less than 2%, the results indicated that the composites are blood compatible. Moreover, the apatite synthesis on the optimized Cu-HAp/CS/PVP composition (80 wt.% of Cu-HAp) in the solution of simulated body fluid (SBF) was indicated by in vitro bioactivity evaluation [87,88,89,90].

The influence of combining the nanoparticles of chitosan and zinc oxide on the composites’ shear bond strength (SBS) was investigated by Mirhashemi et al. for orthodontic bonding applications. Although the highest value of mean SBS was observed in the control group, the lowest value was detected in the composite group including 10% NPs. The index of the adhesive remnant was not varied remarkably through the groups (*p* = 0.823). The combination of chitosan nanoparticles and 1% and 5% zinc oxide did not affect the SBS composition and the achieved SBS amount was the same as that of the control group [82]. In another study, the production of the nanoparticles of CuO and CuO-Chitosan was achieved by co-precipitation. CuO-Chitosan nanoparticles exhibited significant improvement in antidiabetic activity, cytotoxicity, antioxidant and antibacterial activity in vitro, compared to CuO nanoparticles. Additionally, by dentine bonding agents successfully amalgamating the nanoparticles of CuO and CuO-Chitosan, effective therapy versus secondary caries was obtained. Dental adhesive discs were strengthened by CuO-Chitosan nanoparticles that resulted in a remarkable decrease of Streptococcus mutants and Lactobacillus acidophilus. Moreover, the slight change of shear bond strength, solubility plus sustained and slow release profile, absorption of water, and the enhancement of mechanical characteristics were achieved [91,92].

According to the recent research in the field of implant and dental engineering, various chitosan composites for these applications were summarized in Table 1.

## 3. Synthesis Techniques of Chitosan Composites

This paper aimed to share aspects with some available practical techniques for modifying multiple composites of chitosan in the dentistry and implants field. Researchers can be helped by this new trend to opt for the most significant improvement and synthesis techniques for the achievement of desirable features and practical applications. The suitable physicochemical characteristics can be achieved via modification of chitosan structure through enzymatic, chemical, and physical techniques. A number of modification techniques are as follows: modification of plasma-induced, enzymatic modification, radiation-induced modification using radiation-induced grafting, UV, electron beam, and gamma rays, and chemical modification. Different fabrication techniques of micro/nanoparticles of chitosan can be as ionic gelation processes, spray-drying, emulsion-droplet coalescence, and emulsion crosslinking. For example, chitosan hydrogels can be synthesized by covalent crosslinking (interpenetrating polymeric network (IPN) and chemically) and physical association network. This section discusses the synthesis methods for chitosan/carbon-based, chitosan/polymer-based, and chitosan/metal or metal oxide composites [13,102].

### 3.1. Chitosan/Carbon-Based Composites Fabrication Techniques

The insufficient mechanical characteristics and insolubility of chitosan can be modified for biomedical implant applications via carbon-based nanomaterials (CNMs) like reduced graphene oxide (rGO), graphene, GO, and carbon nanotubes for bone cancer treatment. To obtain homogenously dispersed CNMs into the solution of chitosan, the ultra-sonication method can be an excellent procedure. Chitosan-CNT nanofibers are progressed via hydrodynamic focusing, coagulation, and electrospinning techniques, and chitosan-rGO nanofibers can be prepared by the extrusion printing method for cancellous bone regeneration and for biomedical implant applications. For instance, the solution casting procedure is used for the synthesis of chitosan-CNM films. Different techniques are applied to the development of three-dimensional composite scaffolds of chitosan-CNM like freeze-drying and thermally induced phase separation (TIPS) for biomedical implant applications. Some of these synthesis techniques have been used for these mentioned compositions by a number of a researcher like electrodeposition for biomedical implant applications, 3D printing for biomedical application and production possibility of the antimicrobial filaments, freeze-drying for antimicrobial activity, and covalent crosslinking for bone tissue engineering [103,104,105,106,107].

### 3.2. Chitosan/Polymer-Based Composites Fabrication Techniques

Synthesis of chitosan composites combined with polymers, synthetic or natural, is available via various techniques. Fabricating chitosan composites with either synthetic polymers like PCL, PLGA, and PLA or natural polymers such as alginate, silk, gelatin, and collagen are conducted under the freeze-drying condition in the field of bone tissue engineering. Other techniques such as multilayer deposition, compression molding, melt stretching, heat sintering, solvent-extracting, particulate-leaching, plastically compressed self-assembly, dual solid-liquid phase separation, solvent casting, extrusion, electrospinning, and 3D printing are a number of methods for fabrication of chitosan/polymer-based composites [80,108,109,110].

Various investigations have been focused on the fabrication of these compositions. For instance, Sharifianjazi et al. synthesized the composite nanofibers of CS-g-PCL/MBGs and CS-g-PCL/MBGs/Cisplatin in which BGs nanoparticles were prepared using a sol-gel procedure through the molar-ratio chemical composite of 65 SiO_2_-4P_2_O_5_-31CaO. They introduced different values of MBGs and MBGs/Cisplatin (10, and 20 wt.% according to the weight of copolymer) into the solution of CS-g-PCL using further 6 h stirring [111]. Furthermore, in another work, 3D porous nanocomposite scaffolds were fabricated by chitosan, polylactic acid, and calcium phosphate powders, which were synthesized through the freeze-casting procedure. This fabricated composition indicated a pore diameter of 80–380 μm, up to 98% porosity, and improving porosity by reducing calcium phosphate amount. Additionally, the homogeneous distribution was rendered for the pores and calcium phosphate all over the composite structures presenting scaffolds with fundamental features for the engineering of bone tissue [43]. When the development of novel polymer-based restorative dental composites is considered, a new combination of biological and mechanical features is needed.

### 3.3. Chitosan/Metal or Metal Oxide Composites Fabrication Techniques

Metal ions are key elements for bone tissue. Zn^2+^, Cu^2+^, Mg^2+^, and Sr^2+^ are a few metal ions to name. Some techniques such as sol-gel, electrospinning, freeze-drying, and complexation interactions are used to fabricate composites of chitosan-metal [103,112,113,114,115,116]. A chitosan-silver composite scaffold was developed by Vaidhyanathan et al. by utilizing a simple freeze-dry method. Thomas and colleagues generated films of chitosan/silver nanoparticles by employing an in-situ approach. A process in which silver ions in the acidic solution of AgNO_3_ and chitosan were reduced photochemically [117]. In a study by Bhowmick et al. [118] zirconium oxide nanoceramic modified chitosan-based nanocomposite film was formed via incorporating metallic nanoparticles and acid evaporation. Gawad et al. fabricated nanocomposite coatings as a bone implant, which consisted of cobalt nanoparticles (CoNPs) and chitosan (CSNPs) on a bare 316L stainless steel alloy (316L SS). With the electrodeposited technique employing aqueous solutions that contain the solution of 1 mM CoSO_4_, Co nanoparticles were generated. Next, CSNPs were coated onto electrodeposited CoNPs or bare 316L SS alloy. As studied before by Irani et al. via using microwave heating technique, the cobalt ferrite nanoparticles were generated. A porous scaffold of chitosan-calcium-aluminate (CH-AlCa) was developed by Bordini et al., combined with a bioactive dosage of 1α, 25-dihydroxy vitamin D3 (1α, 25VD). These scaffolds were crafted with the aim of being exploited as a bioactive substrate that is able to increase the human dental pulp cells’ (HDPCs) odontogenic potential. By incorporation of the AlCa suspension in a solution of CH under intense agitationg, which was pursued by low-temperature phase separation, a porous structure of CH-AlCa was maintained. The crafted scaffolds’ calcium release, porosity, and architecture were evaluated. Afterward, the synergistic potential of 1 nM 1α,25VD, and CH-AlCa, which were considered by a dose-response assay, for HDPCs seeded materials was assessed [88,119].

## 4. Application of Chitosan Composites for Implant Scaffolds

Altering the surface of metal implants by means of physicochemical, biochemical, or morphological techniques enhances their biocompatibility that leads to a better reaction with living tissue [120]. Metal implants surface modification and coating depositions improve the implant’s performance like biocompatibility and osseointegration [121]. For instance, composite coatings of chitosan, calcium phosphate, and antibiotic gentamicin were obtained by employing the electrophoretic deposition process on the plates of pure titanium. The obtained composite consisting of three various antibacterial active species was effectively evaluated [45].

Employing natural polymers like alginate, collagen, and gelatin in chitosan composites is demonstrated in bone tissue engineering applications. The biodegradable chitosan/alginate scaffolds are indicated to significantly improve mechanical and biological characteristics compared to pure chitosan scaffolds due to their porous network [103]. Further investigations are needed for the biomedical and tissue engineering application of this hybrid polysaccharide. According to previous studies, the application of chitosan scaffold alone is not effective for skin tissue regeneration due to its weak mechanical properties and rapid biodegradation. However, when it’s combined with a suitable modifier like collagen, desirable results were observed in scaffold stability [122,123,124]. The covalent conjugation of chitosan with GO and/or carbon nanotubes creates a scaffold that is more compatible with osteoblast, compared to pure chitosan scaffold. In a study by Ahmed et al., a promising coating for orthopedic implants was obtained, which contained multi-walled carbon nanotubes/chitosan. Two of the most employed bone implants materials and orthopedic coatings are zirconia and Titania. Clavijo and colleagues [125] investigated the chitosan/bioglass/TiO_2_ composite as a coating for 316L stainless steel substrate and improved mechanical properties for biomedical implant applications were obtained. Moreover, in a study by Bartmański et al. [126] high biocompatibility was achieved in a composite of a Ti13Zr13Nb alloy coated with chitosan/nanosilver. Gallium-modified chitosan/poly (acrylic acid) was fabricated on titanium implants by Bonifacio et al. [127], and the antimicrobial properties of the composite were improved. Moreover, the cell proliferation of MG63 osteoblast-like cells was enhanced. Some of the recent studies on the application of chitosan composites for implants are summarized in Table 2.

## 5. Application of Chitosan Compositions for Dentistry

Fluoride can be considered the most critical and well-known dental agent for decades, for dental cavities prevention. Still, there is a concern about dental fluorosis’s high commonness. Manufacturing of enamel demineralization chitosan in vitro interfered via inhibition of mineral elements release from the enamel. The combination of chitosan with propolis displayed a synergic relation for promoting antimicrobial activity. Around the dental braces, enamel demineralization occurred, being effectively prevented by Chitosan-containing dentifrice, during orthodontic treatment. Calcium phosphate carboxymethyl-chitosan composite (CaP-CMCS) material, as well as gypsum-based chitosan (Gp-CT), also is capable of being employed as pulp capping to improve cytocompatibility, differentiation, and proliferation on HDPSCs. The compressive strength was detected to be higher than 600 kPa. This is more than the compressive strength of calcium hydroxide, a typical pulp capping agent. Moreover, the swelling was found to be below 2%, and the rate of degradation was under 10%. Odontoblastic differentiation of pulp stem cells was observed, too. The composite of CaP-CMCS possesses odontogenic potential, biological compatibility, improved mechanical properties, and fast gelation. Therefore, the CaP-CMCS composite fulfills primary key elements of a candidate agent for pulp capping regeneration, which shows biocompatibility, mechanically stability, and rapid curing [45,137].

A porous chitosan/collagen scaffold, chitosan/polymer-based composite, was developed through the freeze-drying method. Next, human dental pulp stem cells (DPSCs) were seeded on the synthesized scaffold surface. The results genuinely revealed gene delivery was enhanced and DPSCs differentiated toward an odontoblast-like phenotype. Shen et al. fabricated chitosan/PLA nanofibers by using emulsion electrospinning. These nanofibers’ application and performance for periodontal bone regeneration were investigated. The results indicated that cell adhesion was improved and osteogenic differentiated to bone marrow stem cells (BMSCs). Moreover, expression promotion of TLR4 of human periodontal ligament cells (hPDLCs) was observed [138,139].

**Figure 4:**
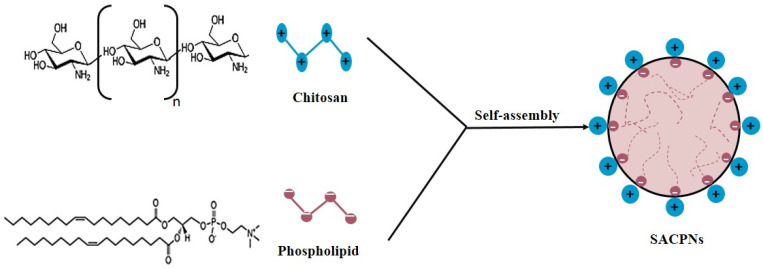
Schematic illustration of the interaction between chitosan and phospholipid, and the SACPNs self-assembly.

The change in pH and calcium ion release in the environment was investigated and measured in a study in the combinations of calcium hydroxide with different mediums, like chitosan, calcium hydroxide, containing distilled water, propylene glycol, and gutta-percha points. The formulation of chitosan exhibited the greatest sustained release of calcium ions. Additionally, compared to other formula mediums, it developed up to 30 day high alkaline pH. Meanwhile, researchers found that the limited drug hydrophobicity on carrier structure is tackled by self-assembly produced chitosan/phospholipid nanoparticles (SACPNs) (Figure 4). The encapsulation efficiency not only is improved but the ability of non-attacking behavior of surrounding enzymes was improved as well. The SACPNs have high encapsulation efficiency and are easy to prepare and mass-produce. Due to their remarkable penetration into gastrointestinal and transdermal mucosa, SACPNs can be employed as systems for multi-functional drug delivery [105].

Via chemical and physical crosslinking, copolymer/graphene oxide/chitosan composite based on poly (N-isopropyl acrylamide) (PNIPAAm) was fabricated. hDPSCs’ osteogenic differentiation to the osteoblasts on the surface of the composite, demonstrated the composite’s favorable features for bone tissue engineering and dentistry [75].

**Figure 5:**
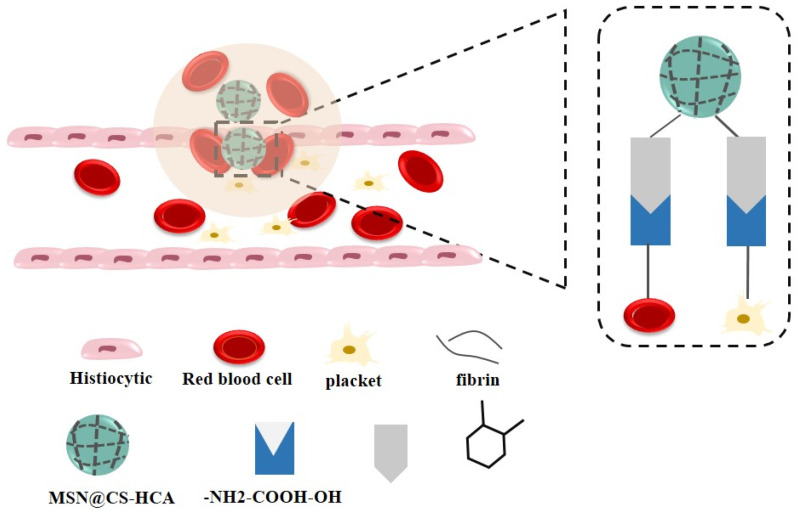
The hemostatic mechanism of MSN@CS-HCA.

Powder hemostatic materials are in high demand in recent years as a result of their portable properties, wide storage temperatures, and prolonged storage time. Mesoporous silica materials gained more interest due to their desirable biocompatibility and significant hemostatic behavior. However, this hemostatic procedure is too plain to fulfill the necessities. Thus, mesoporous silica nanoparticles which were altered by chitosan and hydrocaffeic acid (MSN@CS-HCA) were generated to control hemorrhage rapidly and safely. This is achieved via tissue attachment, the coagulation cascade activation, and finally red blood cells and platelets aggregation (Figure 5). Porous MSN@CS-HCA showed remarkable hemostatic effects coagulation tests both in-vitro and in-vivo [140].

The scarce quality of bone in patients with osteoporosis decreases the dental implant’s osseointegration success rate. The transcription factor to name C-Myb controls gene expression in many cell types as well as the formation of bone. Chitosan-gold nanoparticles could be considered a tool for gene delivery to deliver the C-Myb gene to improve osseointegration in dental implants. The C-Myb gene-containing plasmid DNA is conjugated with chitosan-gold nanoparticles (Ch-GNPs/C-Myb) to inhibit osteoclast genesis and promote osteogenesis. Titanium implants that were coated with Ch-GNP/C-Myb were implanted into mandibles of rats with ovariectomized-induced osteoporosis. Results indicated that the density and volume of newly formed bone were increased. Moreover, osseointegration was observed with dental implants. The bone morphogenic proteins upregulation, and gene regulator RANKL downregulation, were also observed. These can induce osteoclast genesis. JS et al. presented that the Ch-GNPs/C-Myb delivery system, in osteoporotic conditions, facilitates osseointegration of dental implants [45].

## 6. Conclusions and Prospects

In this work, the progress of chitosan and its features for dentistry and biomedical bone implants applications were reviewed. Modification and synthesis of different composites based on chitosan were also studied. These chitosan-based composites were mainly categorized in chitosan/metal-based, metal oxide-based, chitosan/polymer-based, and chitosan/carbon-based composites. The structure, properties, and characteristics of these composites were also introduced. Methods of fabrication and techniques for chitosan synthesis with other materials, as categorized in this study, were also evaluated and investigated using previous studies conducted by other researchers. Moreover, their use in dentistry and implants were surveyed. In the long run, they are favorable biomaterials that can be employed as one of the most eligible materials for dentistry, bone implants, and bone tissue engineering. A number of studies in the field of chitosan-based composites have been carried out; however, many interesting but unexplored compounds and composites have remained yet to be found and studied meticulously in the future and ongoing research. Therefore, they need to be evaluated for the next developments and advancements in the implants and dentistry fabrication.

The primary disadvantage or obstacle is a lack of consensus regarding the physicochemical properties of chitosan, as well as its quality criteria, and significant progress toward this aim has yet to be made. Even the two most essential properties of chitosan, molecular weight and deacetylation level, are still assessed using a variety of analytical methods, with little understanding of the relationships between these methods.

Because the organic component is so important in bone implants, their matrices are frequently hybrid in composition. In viability, mineralization, alkaline phosphate activity, and proliferation experiments, synthetic polymers outperformed natural polymers. Numerous synthetic polymers have been used to fabricate artificial scaffolding materials, but these materials have a number of drawbacks. One of the primary disadvantages of synthetic polymers is that their degradation times are approximately half those of natural polymers.

Additionally, despite CNT’s remarkable mechanical strength and ability to replicate natural bone function, homogeneous CNT dispersion in the chitosan matrix remains a difficulty. CNTs are difficult to distribute and form bundles within the polymer matrix. CNTs need also be extremely pure for biomedical applications, as metal-containing carbon nanotubes are toxic to cells, and producing pure carbon nanotubes is another hurdle. Due to inaccuracies in cytotoxic measurement procedures, the cytotoxicity of nanoparticles is still a subject of debate, and toxicity may vary based on the cell type, nanoparticle purity, and functionalisation.

In summary, the therapeutic and diagnostic potential of chitosan composites in biomedical applications is quite promising. However, a lack of significant knowledge regarding the development of analytical protocols for chitosan quality assessment may delay the momentum of chitin sciences in all sectors of application.

## Figures and Tables

**Table 1:** A list of various chitosan composites for applications of implant and dental engineering.

Composite	The Fabrication Method	In-Vitro Biological Achievement	Application	Ref.
poly (lactic acid-co-trimethylene carbonate)/chitosan (PLA-TMC/Chitosan)	freeze-drying and solvent/nonsolvent sintering method	ALP activity assay and CCK8 cell proliferation assay showed that the scaffolds were conductive to cell adhesion and non-toxic. The scaffolds were potentially usable in bone repair and bone regeneration applications	3D scaffolds	[93]
TiO_2_/Gel-CS (titanium dioxide/gelatin-chitosan) hydrogel	simple chemical approach	The measurements of cytotoxicity and cell attachment by (Live/Dead) and Actin/DAPI staining estimation of hydrogel on cells were preformed. Thus, good biocompatibility, biodegradability and thermal stability of the hydrogel materials have shown that the prepared hydrogel has a good potential for the bone tissue engineering and nursing care applications	orthopedic implants	[94]
collagen, chitosan, and copper-doped phosphate glass composite	co-deposited cathodically	Collagen CS coatings incorporating copper oxide-doped phosphate glass promise to allow the cells to permeate throughout the coatings, create coatings that resemble the extracellular matrix of native bone tissue, enhance the mineralization rate of natural hydroxyapatite, and increase the anti-bacterial properties of the coating. Therefore it will improve surgery procedures and ultimately the quality of life, of patients requiring orthopaedic implants.	orthopedic implants	[95]
AgNP-loaded chitosan–silica class II hybrid	thin film coatings	*Escherichia coli* and Staphylococcus aureus cultures and their biofilm formation were inhibited by all hybrid coatings. Antibacterial effects increased significantly for AgNPs-loaded coatings and appeared to improve with CS content in biofilm assays.	Dental and orthopedic implants	[96]
ternary HAp/chitosan/GO	electrophoretic deposition method (EPD)	There was no Staphylococcus aureus and *Escherichia coli* bacteria growth in broth medium after 1 day and OD600 results at 1 day post inoculation for the 2 wt.% GO addition in coating.	Dental and orthopedic implants	[97]
Poly (lactic acid) (PLA) scaffold surface-modified with chitosan and HAp (PLA/CS/HAp composite)	3D printing	In vitro cell seeding results indicated that bone cells could attach and proliferate at a higher rate on the surface of the CS/HAp modified composite scaffolds compared to the PLA. All the samples were non-toxic to cells and composite scaffolds having CS and HAp on the surface offer better substrate to the cells to adhere, proliferate and migrate.	scaffolds	[98]
chitosan/gelatin layer with silica-gentamicin nanoparticles	electrophoretic deposition, EPD	The good mechanical properties and adherence of the generated coatings on both substrates show the ability for forming a potentially superior bone-to-implant interface for enhancing prosthetic devices performance.	orthopedic implants	[99]
Chitosan/ZnO nanoparticle	deposition	CS/ZnO-coated Ti can be an appropriate material resisting *E. coli* biofilm formation and was compatible with MG-63 cells. Therefore, the coating can be used for orthopedic and dental implant applications.	Dental and orthopedic implants	[100]
Nanocomposite of chitosan-g-poly (acrylamide)/Zn (CPA-Zn)	microwave radiations	In-vitro studies indicated the multifunctional nanocarriers advantage and feasibility for remote-controlled drug release systems	Drug Delivery	[101]

**Table 2:** Some studies about the application of chitosan composites for implants.

Chitosan Composites	Fabrication Technique	Implant	In-Vitro/In-Vivo Achievement	Ref.
HAp-Chitosan Composite Coating	Electrodeposition	Ti_6_Al_4_V alloy	The in-vitro antibacterial and cell viability capabilities of the HNT-CS-MHA composite coating on Ti6Al4V were outstanding; thus, it will serve as an indispensable implant material for orthopedic applications because of its increased corrosion resistance and bioactivity.	[128]
Cellulose nanofiber-reinforced chitosan hydrogel composites	-		Ex vivo research using pig vertebral unit models indicated that implanting CNF-reinforced CHI hydrogels into AF disc lesions aids to disc biomechanics rehabilitation by reaching the functions of a healthy disc.	[129]
Composite of chitosan-gelatin/silica (Si)-antibiotic (gentamicin, Ge)	Spray deposition Electrophoretic deposition.	Commercially pure titanium (cpTi grade 2)	Regarding antibacterial inhibition capabilities, antibacterial activity against both strains (*S. aureus* and *E. coli*) was established using chitosan/gelatin/Si-Ge nanoparticle coatings on titanium substrates, indicating a broad inhibition region surrounding the samples. Both the bare Ti and the coated samples failed to prevent bacterial growth effectively. The presence of silica-based glasses and amorphous silica-based coatings increased cell survival.	[130]
CNT-reinforced chitosan-based ceramic composite	A flexible chemical conversion approach	Pure magnesium	Both biphasic and triphasic composite coatings exhibit improved antibacterial activity when compared to the standard ampicillin. The presence of a greater zone of inhibition shows that CNT-reinforced chitosan-based composite coatings have the potential to limit bacterial growth significantly.	[131]
Hybrid ZnO/chitosan caoting	dip-coating	Surface-modified porous titanium	Cytocompatibility testing revealed that the chitosan/ZnO coating is more compatible with MG-63 cells than pure Ti.	[100]
A chitosan and calcium phosphate-based composite	-	-	At any time, no substantial new bone growth was found in the implants themselves. However, significant new bone growth was detected further away from the drill hole in the rat mandible. The findings indicate that chitosan polymers containing between 50% and 70% DDA boost the normal bone rebuilding mechanism.	[132]
Chitosan/Ce-doped nanobioactive glass (NBG) composite	Electrophoretic deposition	316L stainless steel	When immersed in SBF, the coatings had no cytotoxic impact and formed apatite-like crystals. Additionally, gentamicin was released in a sustained manner by Fickian diffusion. Additionally, drugs/coatings containing NBG demonstrated a greater antibacterial impact than chitosan coatings.	[133]
Polyvinyl alcohol/chitosan/bioactive glass composite	Electrophoretic deposition	316L stainless steel	The disintegration rate of the coats demonstrates that the composite coating with 20% PVA coating has the highest bioactivity and hydroxyapatite formation ability when compared to the 15% and 25% PVA coatings. Similarly, the adhesive test showed that the composite containing 20% PVA is more adhesive than the others.	[134]
Chitosan/gelatin/silica-gentamicin nanocomposite	Electrophoretic deposition	Stainless steel AISI 316L and commercially pure titanium (cp Ti grade 2)	The excellent adhesion and mechanical capabilities of the produced coatings on both substrates reveal their potential for generating a possibly superior bone-to-implant interface, hence improving the function of prosthetic devices.	[99]
Iron oxide-hydroxyapatite-chitosan composite	Electrophoretic deposition	AZ91 Mg alloy	The large increase in iron oxide particles inhibited the growth of microorganisms. The composite coatings also enhanced the apatite mineralization. The hemolysis ratio was less than 5%, indicating that the coatings were naturally compatible with blood. Hydroxyapatite-iron oxide-chitosan composite coatings have a wide range of potential applications in biomedical implant applications.	[135]
The composite of mesoporous bioactive glass nanoparticle (Ag–Sr MBGN) doped with Ag–Sr, and loaded with chitosan/gelatin	Electrophoretic deposition	316L stainless steel	After immersion in SBF, C/G/Ag–Sr MBGN coatings produced a thick HA crystal. Additionally, the coatings demonstrated antimicrobial activity against gram-negative bacteria. The inclusion of Sr to MBGNs decreased Ag’s toxicity.	[136]

## Data Availability

Not applicable.
